# Variant B Cell Receptor Isotype Functions Differ in Hairy Cell Leukemia with Mutated *BRAF* and *IGHV* Genes

**DOI:** 10.1371/journal.pone.0086556

**Published:** 2014-01-30

**Authors:** Nicola J. Weston-Bell, Francesco Forconi, Hanneke C. Kluin-Nelemans, Surinder S. Sahota

**Affiliations:** 1 Tumour Immunogenetics Group, Cancer Sciences Academic Unit, Faculty of Medicine, University of Southampton, Southampton, United Kingdom; 2 Haematology Oncology Group, Cancer Sciences Academic Unit, Faculty of Medicine, University of Southampton, Southampton, United Kingdom; 3 University Medical Center Groningen, Department of Internal Medicine-Haematology, Division of Haematology, Groningen, The Netherlands; University of Manitoba, Canada

## Abstract

A functional B-cell receptor (BCR) is critical for survival of normal B-cells, but whether it plays a comparable role in B-cell malignancy is as yet not fully delineated. Typical Hairy Cell Leukemia (HCL) is a rare B-cell tumor, and unique in expressing multiple surface immunoglobulin (sIg) isotypes on individual tumor cells (mult-HCL), to raise questions as to their functional relevance. Typical mult-HCL also displays a mutated BRAF V(600)E lesion. Since wild type BRAF is a primary conduit for transducing normal BCR signals, as revealed by deletion modelling studies, it is as yet not apparent if mutated BRAF alters BCR signal transduction in mult-HCL. To address these questions, we examined BCR signalling in mult-HCL cases uniformly displaying mutated *BRAF* and *IGHV* genes. Two apparent functional sets were delineated by IgD co-expression. In sIgD^+ve^ mult-HCL, IgD mediated persistent Ca^2+^ flux, also evident via >1 sIgH isotype, linked to increased ERK activation and BCR endocytosis. In sIgD^−ve^ mult-HCL however, BCR-mediated signals and downstream effects were restricted to a single sIgH isotype, with sIgM notably dysfunctional and remaining immobilised on the cell surface. These observations reveal discordance between expression and function of individual isotypes in mult-HCL. In dual sIgL expressing cases, only a single sIgL was fully functional. We examined effects of anti-BCR stimuli on mult-HCL survival *ex-vivo*. Significantly, all functional non-IgD isotypes increased ERK1/2 phosphorylation but triggered apoptosis of tumor cells, in both subsets. IgD stimuli, in marked contrast retained tumor viability. Despite mutant *BRAF*, BCR signals augment ERK1/2 phosphorylation, but isotype dictates functional downstream outcomes. In mult-HCL, sIgD retains a potential to transduce BCR signals for tumor survival *in-vivo*. The BCR in mult-HCL emerges as subject to complex regulation, with apparent conflicting signalling by individual isotypes when co-expressed with sIgD. This suggests the possibility that mutant BRAF by-passes BCR constraints in mult-HCL.

## Introduction

Hairy cell leukaemia (HCL) is a rare B cell leukaemia with characteristic hair-like cytoplasmic projections on tumor cells, displaying distinctive activation markers FMC7, CD103, CD25 and CD11c [1]. A striking feature is the expression of multiple variant surface immunoglobulin (sIg) isotypes on individual tumor cells, in the major subset of disease (mult-HCL) [1].

Seminal studies have established that a functional BCR is essential for survival of normal B-cells, enabling response to cognate antigen or tonic, antigen-independent stimuli [2]. The BCR is a functional complex of sIg flanked by an Igα/Igβ heterodimer with immunoreceptor tyrosine-based activation motifs (ITAMs) in cytoplasmic tails [3]. Cross-linking of BCR by antigen triggers phosphorylation of ITAMs by upstream phosphotyrosine kinases (PTKs) as constituents of the early signalosome. Mammalian wild type BRAF plays an essential role in BCR function, belonging to the RAF family of three cytosolic kinases (A-RAF, B-RAF, C-RAF) that function in the RAS-RAF-MEK-ERK signal transduction, or mitogen-activated protein kinase (MAPK) pathway, a central conduit for survival and proliferation [4]. RAS GTPase proteins activate RAF kinases via dimerization, and the RAF kinases have a restricted substrate specificity to phosphorylate MEK1 and MEK 2 (MEK: MAP/ERK kinase). In turn, the MEK1/2 dual-specificity protein kinases mediate phosphorylation of tyrosine and threonine in ERK1 and ERK2, their only known physiological substrates to date. ERK1/2 on the other hand, have >175 known cytoplasmic and nuclear (including c-Fos, Elk1, Ets1, SP-1) downstream substrates as targets, to signal via diverse pathways to reflect initial stimuli (Review, [4]). Importantly, use of inducible deletion models have shown that wild type BRAF is the critical kinase that transduces BCR signals to activate ERK1/2 using the chicken DT40 B-cell line [5]. Birds encode B-RAF and C-RAF (Raf-1) orthologues but lack a A-RAF equivalent, and in these studies B-raf was identified as the dominant kinase that mediates BCR signals for ERK1/2 activation in B-cells, with an accessory role for Raf-1 [5].

The response of normal B-cells to antigen is stage and context dependent [3], [6]–[9]. Naive B-cells stimulated by antigen undergo apoptosis unless rescued by co-stimulatory signals [7]–[9]. In response to T-cell independent antigens, such as bacterial polysaccharides, specific splenic B-cell subsets are recruited at extrafollicular sites to differentiate locally [6], [10]. In response to T-cell dependent antigens, follicular B-cells are recruited to the germinal center (GC) reaction in secondary lymphoid follicles, to initiate somatic hypermutation (SHM) of Ig variable (*IGV*) region genes [11]. Deletional isotype class switch recombination (CSR) can follow, to in general yield B-cells expressing a single isotype switched IgH. Both SHM and CSR depend on the enzyme activation induced cytidine deaminase (AID) [12]. Cessation of GC events generates durable IgM+ or isotype class switched memory B-cells that have improved affinity for antigen [11], and generally express CD27 [13].

Whether the BCR plays a comparable role in sustaining survival in malignant B-cells is less well characterized and remains a central question in understanding origins and progression of mature B-cell neoplasms. A role for antigen recognition by the BCR in selection of the tumor clone or driving persistence via the BCR has been investigated in diverse B-cell tumors: it is clear that signalling via the BCR is implicated, as recent data from genome sequencing highlights abnormalities in key downstream pathways such as CARD11 in DLBCL, and constitutive activation of the PI3K pathway in lymphoma, or that the BCR itself is selectively modified to promote tumor: niche interactions, as in follicular lymphoma to potentially bypass a requirement for antigen (Review, [14]).

In mult-HCL, the functional role of BCR in primary tumor cells has not been examined to date. Most mult-HCL tumors exhibit SHM with a low level of on-going mutations and *AID* expressed, implicating initial contact with antigen via the BCR [1], [15], [16]. The pathway(s) that progress transformation however are as yet not fully mapped. Interestingly, whole exome sequencing in typical HCL identified mutant *BRAF* V(600)E as almost universal in this tumor type [17]. There are also cases that lack this mutation, frequently expressing *IGHV4-34* genes, in which pathogenesis may differ [18]. Consistent with mutant *BRAF* V(600)E, ERK1/2 is constitutively activated and levels of phosphorylated ERK (p-ERK) are raised in HCL [19], [20]. As there is an essential requirement for wild type BRAF in transducing BCR signals [5], it remains to be established how mutant BRAF can affect BCR function in HCL, given the requirements for dimerization of wild type BRAF for ERK1/2 activation [4], and whether ERK1/2 activation can be enhanced by functional BCR signals for downstream signals in HCL.

Here, we report on BCR function in mult-HCL, focusing on the role of individual isotypes and their relevance to tumor persistence, in cases uniformly displaying mutant *BRAF* V(600)E and mutated *IGHV* genes. This study extends our preliminary observations on BCR function in HCL [21].

## Materials and Methods

### Ethics

Ethical approval was obtained from institutional bodies. The HCL samples were stored and provided by Professor H. Kluin-Nelemans, and have been approved for use by Ethical Review by the NHS Health Research Authority UK, NRES Committee South Central under REC M228/02/t. The HCL samples comprise an existing holding collected in 1980–1990 and for which consent is waived by the Human Tissue Authority.

### Patient Samples

Diagnosis of typical HCL disease was established by clinical criteria, morphology of neoplastic cells in blood, histology of bone marrow and spleen, cytochemical analysis, and immunophenotype (CD11c^Hi^/CD19^+^/CD22^+^/CD25^+^) [22]. Patients underwent splenectomy, and disaggregated splenocytes were stored in liquid N_2_. Unselected cases were analysed. Frozen splenocyte samples were thawed, washed, resuspended and allowed to stabilise prior to use (full details are given in Supporting Information; File S1).

### Immunophenotyping

Multiparameter flow cytometry (FC) with fluorochrome conjugated F(ab’)_2_ antibodies was used to immunophenotype hairy cell (HC) subpopulations in individual tumors [15], [16] (detailed protocol is described in File S1).

### Analysis of *IGHV* Genes and *BRAF* V(600)E Mutation

Tumor-derived *IGHV* genes and the *BRAF* V(600)E mutation were identified as reported [15], [16], [23]. DNA sequencing spectra delineated between monoallelic and biallelic *BRAF* V(600)E mutations [23].

### BCR Induced Intracellular Calcium Flux

Washed cells were loaded with the free Ca^2+^ sensor dye Fluo-3AM and labelled with anti-CD19/CD11c antibodies for FC, and stimulated with isotype specific or sIgL antibodies for tracing Ca^2+^ flux (detailed protocol is described in File S1).

### Phosflow Protocol for Measurement of BCR Induced ERK Phosphorylation

This protocol utilized a mild alcohol permeabilization after stimulating HCs with goat F(ab’)_2_ anti-human IgA, IgD, IgG, IgM, kappa or lambda to assay ERK1/2 phosphorylation using a specific α-phosphoERK1/2-alexafluor488 antibody. Data was acquired on CD19^+^CD11c^Hi^ gated lymphocytes for analysis of shifts in phosphoERK fluorescence by FC (detailed protocol is described in File S1).

### BCR Endocytosis

Two methods were employed: either cells were stained with a FITC-labeled rabbit F(ab’)_2_ anti-goat F(ab’)_2_ and a secondary antibody to detect remaining goat F(ab’)_2_ on the cell surface, or stained with rabbit F(ab’)_2_ anti- human Ig antibodies, as for immunophenotyping, to allow analysis of changes in paired IgH and IgL chains (detailed protocol is described in File S1).

### Induction of Intracellular Calcium Flux following BCR Endocytosis

Endocytosis of sIg was induced as above, with pre-binding of goat F(ab)_2_ anti-IgH or IgL, then incubated at 37°C, washed and loaded with fluo3-AM, followed by staining with α-CD19-V450 and α-CD11c-APC (detailed protocol is described in File S1).

### Induction of Apoptosis following BCR Stimulation

Splenocytes were stimulated with soluble goat F(ab)_2_ α-human IgA, IgD, IgG, IgM, kappa or lambda antibody respectively or with immunobead-conjugated anti-human IgA, IgG or IgM coated immunobeads in medium for 2/3 hours at 37°C. Induction of apoptosis was measured using Apostat or Apo2.7-PE as detailed in File S1.

### Analysis of Proliferation following BCR Stimulation

HCL cells were stimulated with F(ab)_2_ α-human IgA, IgD, IgG, IgM, kappa or lambda at 37°C and proliferation assessed using a metabolite capture Cell Proliferation Kit II (XTT) (detailed protocol is described in File S1).

## Results

### Phenotype, *BRAF* and *IGHV* Mutational Status

We utilized CD19^+^CD103^+^CD11c^Hi^ expression to gate specifically on HCs, and F(ab’)_2_ antibodies to exclude non-specific FcR binding. CD103 expression correlated tightly with CD11c^Hi^ expression (Fig. S1), permitting use of CD19^+^CD11c^Hi^ FC gates to delineate tumor cells and exclude non-tumor B-cells (n = 10 mult-HCL cases). To confirm this, tumor cells obtained in 2/2 cases by flow sorting using CD19^+^CD103^+^CD11c^HI^gates achieved purities of 91.1–99.8%, and in these the tumor-derived *IGHV* marker gene was found in >98% of all clones examined in each case (data not shown). Splenic tumor burden ranged from 52–90% using these markers (Table S1 in File S1).

The mult-HCL phenotype was confirmed by examining single HCs by multiparameter FC (Fig. S1; Table S1 in File S1). These data delineated 2 subsets by phenotype, differing by IgD co-expression, with sIgκ or sIgλ or both light chains expressed.

In IgD^+ve^ mult-HCL, with sIgD expressed in >90% tumor cells, each case also displayed multiple isotypes, both pre-switched and post-switched (n = 4 cases; Table S1 in File S1). Patterns of co-expressed isotypes on individual HCs varied. In IgD^+ve^ Cases 7 and 9, IgD/IgG/IgM/kappa were co-expressed on >90% of cells. In IgD^+ve^ Case 13, IgD/lambda were expressed on all cells with subpopulations expressing IgA (26%), IgG (95%), IgM (89%) and kappa (88%), and similarly, in IgD^+ve^ Case 6 all cells expressed IgD/lambda with subpopulations expressing IgA (34%), IgG (62%), IgM (20%) and kappa (72%).

In IgD^−ve^ mult-HCL, tumor cells displayed IgG/M or IgG/M/A isotypes but clearly lacked surface IgD expression (n = 6 cases; Table S1 in File S1). In IgD^−ve^ Cases 2, 3 and 4, IgG/kappa was expressed on 100% of cells, with a subpopulation of cells co-expressing IgM (50–90%). In IgD^−ve^ Case 5, 100% of cells expressed IgA/IgG/lambda, with subpopulations of cells co-expressing IgM (48%) or kappa (86%) respectively. IgD^−ve^ Case 10 expressed IgG/lambda on 100% of cells, with subpopulations of cells co-expressing IgA (72%) and IgM (18%), respectively (Table S1 in File S1; Fig. S1). In IgD^−ve^ Case 11 IgG/kappa was expressed on all cells, with IgA and IgM co-expressed on 12% and 13% of cells, respectively.

Dual light chain expression was observed in 3 mult-HCL cases (Cases 5,6,13; Table S1 in File S1), confirming our previous observations [24]. In 8/8 cases examined, CD27 expression was absent on gated tumor cells (Table S1 in File S1), as previously reported [25].

The *BRAF* V(600)E mutation was verified in 10/10 cases (Table S1 in File S1) by amplification and Sanger DNA sequencing [23]; in each case, the mutation was found to be monoallelic. *IGHV* gene analysis delineated 10/10 cases as mutated (98.6–92.2% homologous to germline), with clonally-derived multiple isotype switched transcripts in the tumor population (Table S1 in File S1).

### BCR Induced Ca^2+^ Flux

**Figure 1:**
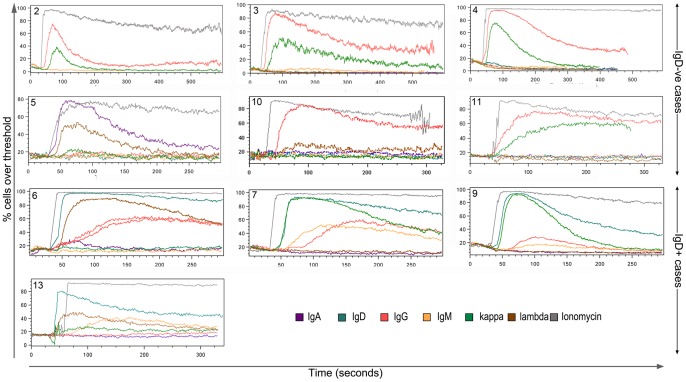
BCR induction of calcium flux is functionally intact in both IgD^+^ and IgD^−ve^ mult-HCL. Ca2+ flux was measured in response to goat F(ab’)_2_ anti-IgH or anti-IgL mediated BCR ligation using the cytoplasmic calcium-indicator dye Fluo3-AM. Unstimulated cells were recorded for 30 seconds prior to addition of goat F(ab’)_2_ polyclonal anti-Ig. Stimulated cells were then tracked for 5 minutes: cells were gated first on FSC/SSC, then as CD19^hi^CD11c^hi^ for cases 1–9 or as CD19^hi^CD11c^hi^CD103+ for cases 10–13 prior to analysis of Fluo3-AM fluorescence. The plots here show % cells over threshold where the threshold is the 85^th^ percentile of unstimulated cells. Ionomycin was used as control to achieve maximal Ca^2+^ flux.

BCR stimulation triggers tyrosine kinase phosphorylation events and activation of phospholipase C to release intracellular Ca^2+^ from the endoplasmic reticulum into the cytosol, and this can be assayed using cytoplasmic dyes (fluo3-AM) that fluoresce in the presence of free Ca^2+^. BCR mediated Ca^2+^ flux was examined in HCs gated by CD19^+^CD11c^Hi^ (7/10) or CD19^+^CD103^+^CD11c^Hi^ (3/10) expression. Initial studies indicated that anti-CD19/CD103/CD11c antibodies respectively did not induce Ca^2+^ flux (data not shown). All cases (10/10) revealed functional BCRs that transduced Ca^2+^ flux (Fig. 1). However, striking differences emerged between the IgD^+ve^ and IgD^−ve^ subsets. In IgD^+ve^ cases (6, 7, 9 and 13), invariably more than one sIgH isotype triggered Ca^2+^ flux (Table 1). In these cases, IgD produced the predominant response, while IgG and/or IgM were capable of stimulating lower levels of flux. In contrast, in IgD^−ve^ cases (2, 3, 4, 5, 10 and 11), only signals via a single switched isotype (IgG/A) induced flux but not the co-expressed IgM (cases 2, 3, 4, 5, 10 and 11) or IgG (case 5) or IgA (cases 10 and 11) (Table 1). While, in some IgD^−ve^ cases this lack of response may be linked to lower levels of surface expression (IgA in case 11; IgM in cases 5, 10 and 11), this does not account for lack of function in more highly co-expressed isotypes (IgM in cases 2, 3 and 4; IgG in case 5; IgA in case 10). These non-functional isotypes in IgD^−ve^ cases are co-expressed at comparable levels to functional isotypes in IgD^+ve^ cases. It may instead point to a selected feature that distinguishes the two mult-HCL subsets.

**Table 1:** Mult-HCL immunophenotype and isotype function in transducing Ca2^+^ flux to ERK 1/2 phosphorylation.

sIg status	Case	sIg Phenotype*						Ca2+ flux#						ERK phosphorylation$					
		A	D	G	M	K	L	A	D	G	M	K	L	A	D	G	M	K	L
**D-ve**	**2**	–	–	+++	++	+++	–	–	–	+++a	–	++a	–	ND	ND	++a	**–**	+a	ND
**D-ve**	**3**	–	–	+++	++	+++	–	–	–	+++a	–	++a	–	ND	ND	++a	–	+a	ND
**D-ve**	**4**	–	–	+++	++	+++	–	–	–	+++a	–	++a	–	ND	ND	ND	ND	ND	ND
**D-ve**	**5**	+++	–	+++	+	++	+++	++a	–	–	–	–	++a	++d	ND	ND	ND	–	++d
**D-ve**	**10**	++	–	+++	+	–	+++	–	–	+++a	–	–	+a	ND	ND	ND	ND	ND	ND
**D-ve**	**11**	+	–	+++	+	+++	–	–	–	+++a	–	+++a	–	ND	ND	ND	ND	ND	ND
**D+ve**	**6**	+	+++	++	+	++	+++	+c	+++b	++c	–	–	+++b	−/+c	+++a	+/−c	–	–	+a
**D+ve**	**7**	–	+++	+++	+++	+++	–	–	+++b	+c	+/−c	+++b	–	ND	+++a	+/−c	–	++a	ND
**D+ve**	**9**	–	+++	+++	+++	+++	–	–	+++a	++c	++c	+++a	–	ND	+++a	++c	+/−c	+++a	ND
**D+ve**	**13**	+	+++	+++	++	++	+++	–	+++b	(+)c	++c	(+)c	++b	–	++b	+c	+a	–	++a

* Surface immunoglobulin (sIg) phenotype data is reproduced from Table S1 to allow for ease of comparison when evaluating Ca2+ and ERK phosphorylation data.

# Intensity of Ca^2+^ flux responses is denoted by +/−; – <5% of cells responded, +10–35% of cells responded, ++35–70% of cells responded,+++>70% of cells responded. Flux patterns are denoted by lower case letters; a-fast transient, b-fast persistent, c-slow biphasic/persistent, d-fast biphasic.

$ For ERK phosphorylation signal intensity is indicated by −/+/++/+++ and type of signal by lower case letters as for Ca^2+^ flux: a- fast transient, b- fast persistent, c- slow biphasic/persistent, d- fast biphasic.

This functional dichotomy was also accompanied by marked differences in the patterns of Ca^2+^ flux observed. When IgD was co-expressed, it tended to trigger persistent responses accompanied by transient responses from the less prominent isotype(s) (Fig. 1), whereas all flux responses in IgD^−ve^ mult-HCL were of the fast transient type (Table 1). In normal circulating B cells, IgD or IgM stimuli induce persistent calcium flux (data not shown).

Overall, measurements of Ca^2+^ flux in mult-HCL were carried out in 6/6 IgD^−ve^ and 4/4 IgD^+ve^ cases, confirming observations in ‘biological’ replicates. Whether these patterns are consistently observed in each IgD subset awaits further study when larger cohorts are examined.

In relation to dual light chain expression, in the IgD^+ve^ mult-HCL Case 13 a low level of Ca^2+^ flux was observed via the second light chain κ in addition to the predominant λ response; in Case 5 and 6, this cationic flux was only induced by the λ chain (Table 1).

### BCR Induced Downstream ERK Phosphorylation

**Figure 2:**
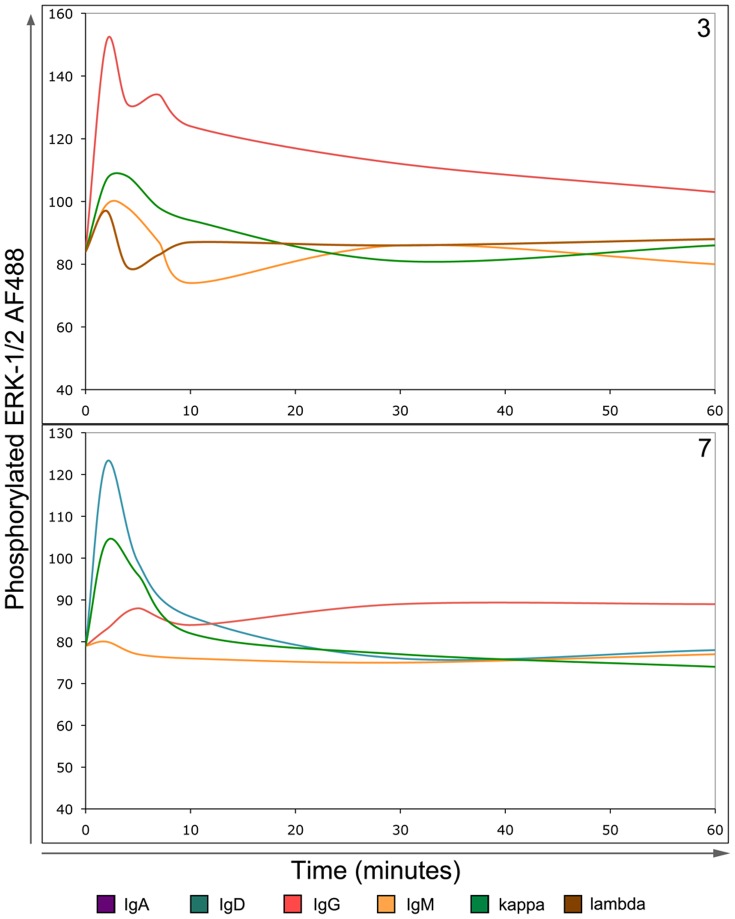
BCR-induced signals trigger phosphorylation of ERK1/2 in mult-HCL. The BD phosflow assay system was used to measure phosphorylation of ERK1/2 following BCR stimuli. Cells were stimulated using goat F(ab’)_2_ anti-Ig antibodies for indicated times, fixed, permeabilised and CD19^HI^CD11c^HI^ lymphocytes examined for intensity of phosphorylated ERK1/2 staining. Patterns of ERK1/2 phosphorylation reflected induced calcium flux. Two representative examples are shown. Upper Panel: In IgD^−ve^ mult-HCL Case 3 anti-IgG or κ stimuli induce Ca^2+^ flux and ERK1/2 phosphorylation. Notably, sIgM shown not to induce Ca2+ flux did not trigger ERK1/2 phosphorylation. Lower panel: In IgD^+^ mult-HCL Case 7, ERK1/2 phosphorylation patterns again reflected Ca2+ flux, both in intensity and type of signal. For example IgG-induced calcium flux was of low intensity and slow (Table 1), which is mirrored by the ERK1/2 phosphorylation kinetics observed. Both panels: Note that at early time points (1–2 minutes) a non-specific response is commonly observed in response to addition of antibody, as can be seen with anti-IgM and anti-λ negative controls in Case 3.

Downstream BCR signals initiate ERK1/2 phosphorylation via the MAPK pathway to mediate cellular responses. ERK1/2 phosphorylation was examined in mult-HCL able to induce Ca^2+^ flux by crosslinking BCR. In 7/7 cases examined from both subsets, ERK1/2 phosphorylation was restricted to the IgH isotype or single IgL that transduced Ca^2+^ flux via the BCR (Table 1; Fig. 2). Negative control isotypes or IgL chosen as they did not induce Ca^2+^ flux failed to phosphorylate ERK1/2, demonstrating specific functional coupling (Table 1; Fig. 2). The kinetics of ERK1/2 phosphorylation as compared to Ca^2+^ flux varied. Although IgD-induced Ca^2+^ flux signals were generally persistent (marked with ‘b’, Table 2), the corresponding ERK1/2 phosphorylation response tended to be transient (marked with ‘a’, Table 2). In IgD^−ve^ Cases 2, 3 and 5 where all calcium flux responses had been fast transient (marked with ‘a’, Table 2), ERK phosphorylation was predominantly fast transient (Table 2; Fig. 2, upper panel).

**Table 2:** Linking anti-sIg stimuli in HCL to downstream effects on tumor apoptosis.

					% Cells apoptotic			
	sIg				Assay 1		Assay 2	
Case	Status	sIg Phenotype	Functional BCR sIg	StimulatingAntibody	2 hours	3 hours	2 hours	3 hours
3	D-ve	IgG, IgM, kappa	IgG, kappa	CON (IgD)	ND	5.65	ND	7.79
				IgG	ND	25.43***	ND	33.70****
				kappa	ND	23.40****	ND	23.63****
5	D-ve	IgA, IgG, IgM, kappa, lambda	IgA, lambda	CON (IgD)	6.93	6.28	ND	ND
				IgA	19.45****	12.80****	ND	ND
				lambda	14.70****	16.83****	ND	ND
10	D-ve	IgA, IgG, IgM, lambda	IgG, lambda	CON (IgD)	9.13	9.50	12.60	9.50
				IgG	21.48****	29.20****	29.23***	32.10***
				lambda	23.98****	23.10***	24.45***	28.75****
				CON^#^10 (IgA)	ND	14.02	ND	10.40
				IgG^#^10	ND	17.82*	ND	16.52***
				CON^#^20 (IgA)	ND	14.04	ND	13.58
				IgG^#^20	ND	23.68***	ND	18.68***
11	D-ve	IgA, IgG, IgM, kappa	IgG, kappa	CON (IgD)	20.78	21.10	20.60	ND
				IgG	35.53****	34.08****	32.83****	ND
				kappa	39.65***	41.53****	38.55**	ND
13	D+ve	IgA, IgD, IgG, IgM,kappa, lambda	IgD, IgM, IgG, lambda,kappa	CON (IgA)	ND	5.63	ND	4.58
				IgD	ND	5.93^ns^	ND	4.33^ns^
				IgM	ND	8.95***	ND	5.50***

Data were analysed in PRISM using Student’s t-test with Welch’s correction. P-values are denoted as: ns>0.05,

* 0.01–0.05,

** 0.01–0.001,

*** 0.001–0.0001,

**** <0.0001.

### BCR Endocytosis

**Figure 3:**
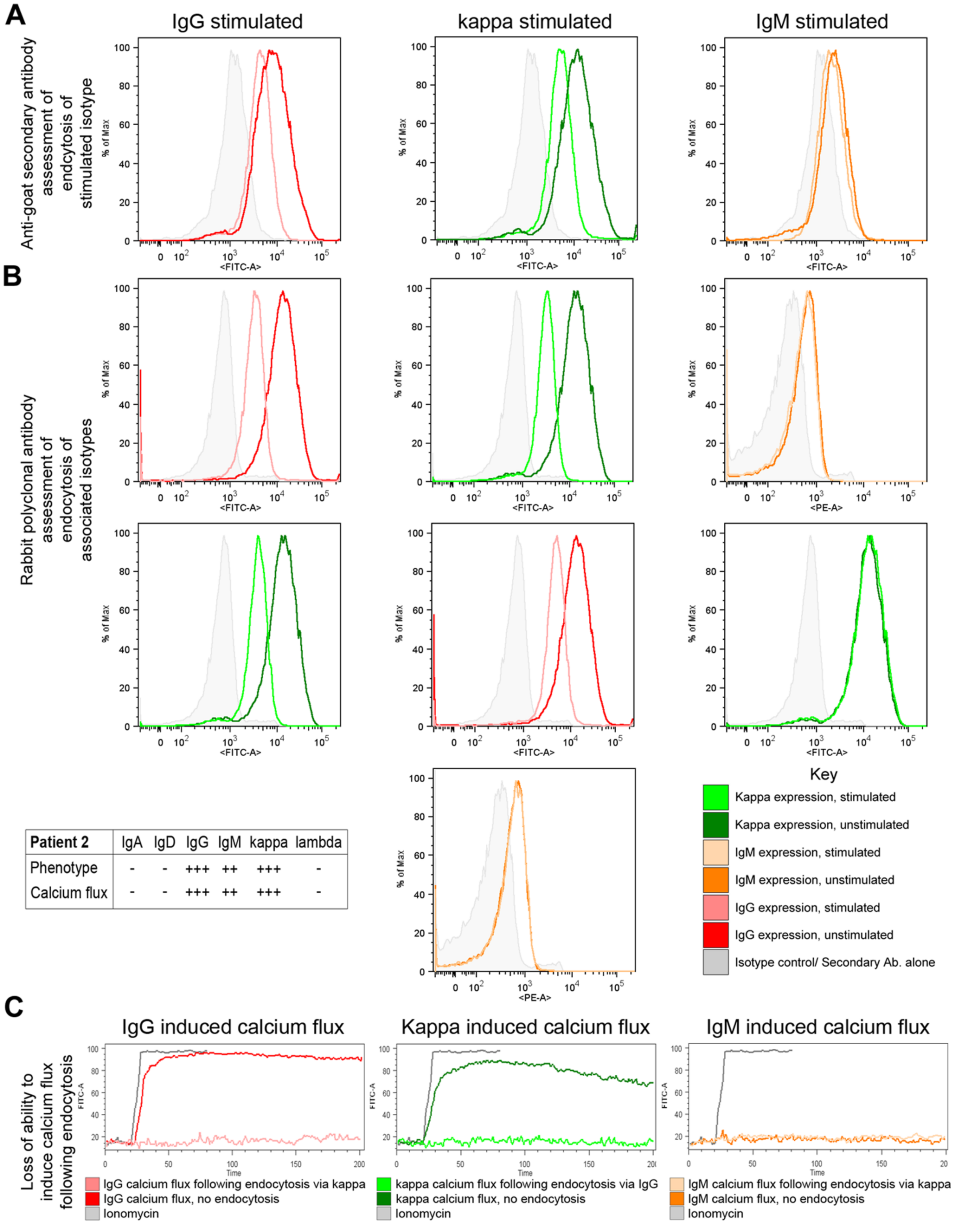
Specific anti-sIg stimuli mediate endocytosis of functional BCR in IgD^−ve^ mult-HCL. A representative example is shown. Cells were stimulated with goat F(ab’)_2_ anti-Ig antibodies for 1 hour either at 37°C to allow apoptosis or at 4°C to prevent it. Changes in stimulated sIg expression levels were then measured by secondary staining using mouse anti-goat F(ab)_2_ (A). Changes in expression of paired sIg were measured using rabbit F(ab’)_2_ anti-Ig (B). Loss of functional sIg was further confirmed by measuring induction of Ca^2+^ flux following endocytosis (C). Using the anti-goat secondary antibody method stimulation of sIgG (A, left panel) and sIgκ (A, middle panel) in Case 2 resulted in reduction of surface expression of these two molecules. Stimulation of non-functional sIgM in this patient did not result in endocytosis (A, right panel). Using the rabbit F(ab’)_2_ staining method stimulation of IgG (B, left panel) in Case 2 resulted in loss of sIgG (B, top left panel) and of sIgκ (B, lower left panel). Stimulaton of sIgκ also triggered specific patterns of endocytosis, as shown by loss of sIgG and sIgκ expression, but no change in sIgM expression (B, centre panel). Stimulation of non-functional sIgM generated no changes in sIgM expression, or in sIgκ expression (B, right panel). Inset shows a Table of the sIg phenotype and functional isotypes in Case 2 included as a reference to correlate data (B, lower left). Following endocytosis of BCR by stimulation with anti-Igκ, the ability to induce Ca^2+^ flux via sIgG was completely ablated (C, left panel). Similarly following endocytosis of BCR with anti-sIgG, the ability to induce Ca^2+^ flux via κ was also negated (C, middle panel). No Ca^2+^ flux was induced through dysfunctional sIgM either before or after endocytosis stimuli (C, right panel).

Normal B cells endocytose the BCR following cognate antigen recognition to mediate processing and presentation of antigen [26]. In mult-HCL, BCR endocytosis following goat F(ab’)_2_ anti-human IgH/IgL specific stimuli was evaluated by two different methods. Firstly, by changes in levels of bound stimulating antibody following endocytosis were measured by staining with a FITC-labelled rabbit F(ab’)_2_ anti-goat F(ab’)_2_ secondary antibody (Fig. 3 A and Fig. S2 A, top panels). Secondly, by changes in levels of expression of the stimulated IgH isotype/IgL and of sIgH/IgL pairings by specific staining with FITC- and PE-labeled rabbit F(ab’)_2_ anti-human Ig antibodies (Fig. 3 A and S2 A, middle and lower panels). The latter method has the disadvantage that when staining for the same IgH/IgL as stimulated, stearic hindrance may exaggerate apparent loss of expression. This stearic hindrance however is avoided with the anti-goat secondary antibody method. Where feasible, Ca^2+^ flux was also evaluated following endocytosis.

Two IgD^+ve^ mult-HCL cases were examined. In Case 13 (sIgA^+^, D^+++^, G^+++^, M^++^, κ^++^, λ^+++^; Table S1 in File S1), anti-IgD stimuli reduced sIgD and sIgλ expression via endocytosis, but had no effect on sIgκ (Fig. S2 A, first column data). Anti-sIgλ stimuli triggered a reduction in sIgD, sIgM and sIgλ levels (Fig. S2 A, second column data), and a small reduction in sIgG expression but no change in sIgA (data not shown). This effect on sIgG endocytosis, in relation to high levels of expression and relatively low levels of Ca^2+^ flux and ERK1/2 phosphorylation following anti-sIgG perturbation (Table 1), suggests either a restricted functional coupling or possibly a gradual loss of sIgG function. In addition, anti-sIgM stimulation partly reduced sIgM expression but had no measureable effect on slgλ or sIgκ (Fig. S2 A, third column data) suggesting that in mult-HCL, pairings of sIgH isotype occur with specific sIgL when dual light chains are expressed on HCs and that only one sIgL chain retains functionality. However, sIgA expression is relatively low on these HCs and insufficient levels for effective cross-linking may explain the lack of endocytosis and Ca^2+^ flux (Fig. S2 A; fourth column data). Further analysis of Case 13 indicated that BCR endocytosis using specific anti-sIg stimuli affected signal transduction to induce Ca^2+^ flux, revealing fully functional internalisation events. Anti-sIgD treatment completely ablated Igλ induced Ca^2+^flux, while endocytosis of sIgM greatly reduced it (Fig. S2 B, centre panel). This confirms functional integrity of sIgM expression in IgD^+ve^ mult-HCL. Induction of endocytosis by anti-Igλ stimuli reduced measurable Ca^2+^ flux via sIgD (Fig. S2 B, left panel), but completely ablated IgM-induced flux (Fig. S2 B, right panel). This observation indicates kinetic differences in loss of functionality of sIgH and sIgL, depending on specific endocytosis triggers.

Comparable patterns of BCR response were observed in IgD^+ve^ mult-HCL Case 6 (sIgD^+++^, G^++^, A^+^, M^+^, κ^++^, λ^+++^) where, due to limited availability of material, a reduced analysis was performed. Endocytosis by anti-IgD stimulation resulted in a reduction in sIgD/sIgλ, and a loss of λ-induced Ca^2+^ flux, whereas the corresponding anti-λ mediated endocytosis resulted in reduction of sIgλ/sIgD expression and reduction in IgD induced Ca^2+^ flux (data not shown).

In IgD^−ve^ mult-HCL Cases 2, 3 and 4 (each with phenotype sIgG^+++^, M^++^, κ^+++^), endocytosis of sIgG and sIgκ followed BCR engagement with anti-sIgG, and endocytosis triggered via sIgκ reduced sIgG and sIgκ expression, but had no effect on levels of sIgM expression in 3/3 cases (Fig. 3 A and B; Table 1). This confirmed a non-functional, immobilized state for sIgM in IgD^−ve^ mult-HCL, as Ca^2+^ flux was also absent via the sIgM isotype (Table 1). The ability to induce Ca^2+^ flux through functionally paired sIgGκ was greatly reduced following endocytosis of sIgG or sIgκ (Fig. 3 C for Case 2).

### BCR Mediated Selective Apoptosis

**Figure 4:**
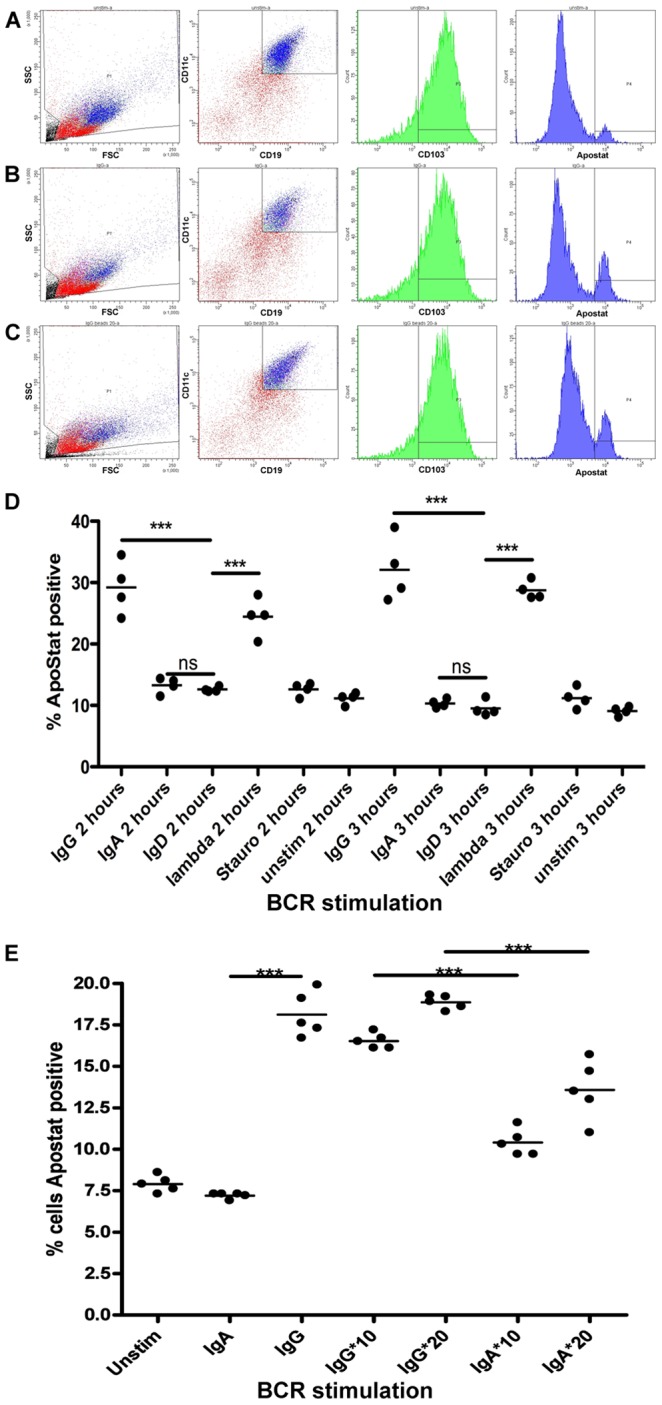
Signalling via BCR using soluble or immobilised anti-sIg antibodies induces apoptosis in IgD^−ve^ mult-HCL. Tumor cells were stimulated with goat F(ab’)_2_ anti-Ig antibodies at 20 µg/ml or immobilised mouse anti-Ig using immunobeads at 10 or 20 µg/ml (represented as IgG*, IgA*) for the indicated times at 37°C and early onset apoptosis measured using the Apostat assay. Panels A–C show as an example FC plots from Case 10 (sIgA^++^, IgG^+++^, IgM^+^, lambda^+++^, with functional BCR isotypes IgG and λ) revealing increased apoptosis (% Apostat positive CD19^HI^CD11c^HI^CD103^+^ HCs) in response to soluble (Panel B) and immobilised (Panel C) anti-IgG as compared to response to non-functional isotype (anti-sIgA) as control (Panel A). Panel D shows representative data from 1 of 2 replicate experiments using soluble anti-sIg stimuli and increase in apoptosis in response to both functional sIgG and λ mediated signals as compared to a negative control isotype that is not expressed (anti-sIgD). Staurosporin, included as a potential agent for apoptosis, had no measurable apoptotic effect at time of assay. Panel E shows data from 1 of 2 replicate experiments comparing soluble –vs- immobilised anti-sIg stimuli for 3 h (data read-outs shown in Table 2). The data shows that immobilised anti-sIg stimulation also triggers apoptosis (at both 10 and 20 µg/ml) as compared to the immobilised non-functional isotype control (anti-sIgA). Data were analysed in PRISM using Student’s t-test with Welch’s correction. P-values are denoted as: ns>0.05, *0.01–0.05, **0.01–0.001, ***0.001–0.0001, ****<0.0001.

In each of 3 IgD^−ve^ mult-HCL cases (5,10,11), stimulation via functional sIgG or light chain with soluble goat F(ab’)_2_ anti-human antibody induced significant levels of apoptosis as compared to dysfunctional sIg isotype, which were used as controls (Fig. 4; Table 2). In IgD^−ve^ mult-HCL Case 10, where material was available, we investigated the effect of immobilised antigen using antibodies coupled to beads (Fig. 4 E). Surrogate antigen in this format, which maximises cross-linking to limit endocytosis, also triggered apoptosis but not above levels seen with soluble antibody (Fig. 4 D; Table 2). In 2 IgD^−ve^ mult-HCL cases (3,10), an additional XTT based assay for proliferation showed no such effects when stimulated with functional anti-sIg antibodies (Fig. S3).

**Figure 5:**
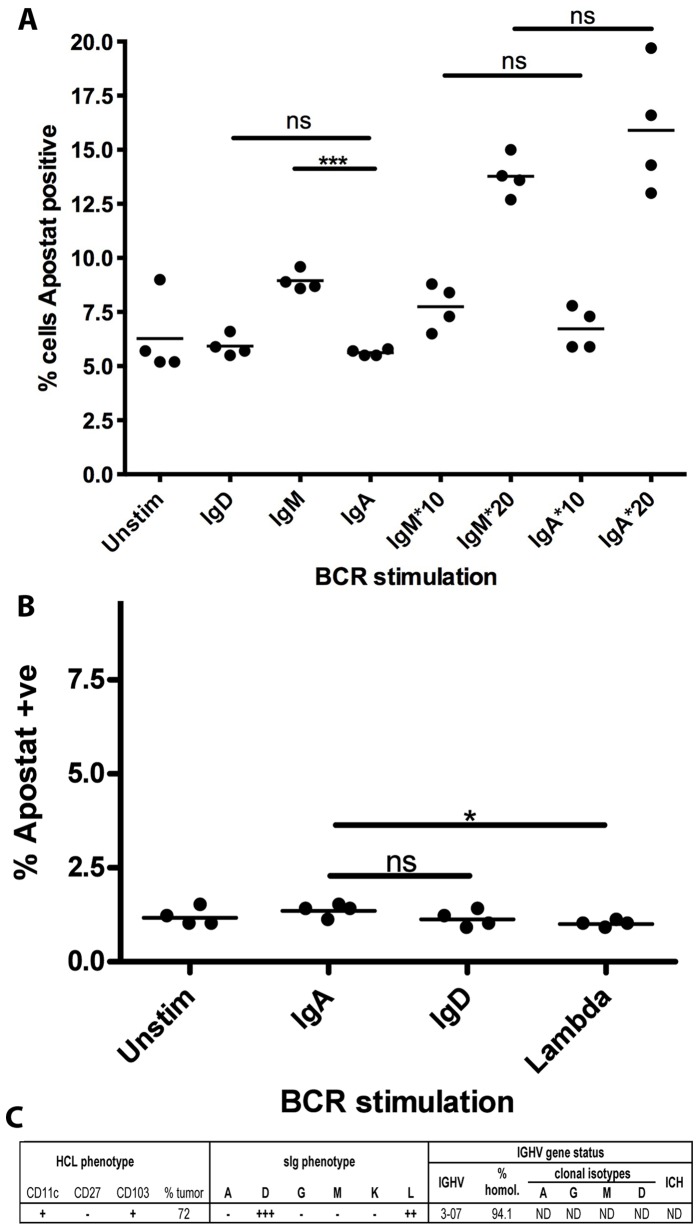
In IgD^+^ mult-HCL and IgD^+^ s-HCL anti-sIgD stimuli do not trigger apoptosis. For IgD+ mult-HCL Case 13 (functional isotypes for ERK1/2 phosphorylation: D^++^, G^+^, M^+^, λ^++^) material was available to evaluate stimulation with soluble goat F(ab’)_2_ anti-sIgD/λ antibodies or anti-sIgA as control (isotype not functional). Panel A shows representative data from 1 of 2 replicate experiments assaying apoptosis in CD19^HI^CD11c^HI^CD103^+^ tumor cells following stimulation. Anti-sIgD stimulation did not induce apoptosis as compared to the non-functional isotype control. Stimulation with soluble anti-sIgM did induce a low but significant increase in apoptosis as this isotype is functional. This lack of apoptotic effect in response to IgD stimulation was further investigated in a single sIgD+ve s-HCL Case 15 (phenotype summarised in Panel C). Stimulation via sIgD or slgλ in this s-HCL case did not induce apoptosis of CD19^HI^CD11c^HI^CD103^+^ tumor cells either in response to soluble (Panel B) or immobilised anti-sIg (data not shown). Data were analysed in PRISM using student’s t-test with Welch’s correction. P-values are summarised as ns>0.05, *0.01–0.05, **0.01–0.001, ***0.001–0.0001, ****<0.0001.

In IgD^+ve^ mult-HCL analysis of apoptosis post-BCR stimuli was restricted to a single case due to sample availability. For Case 13, BCR retained functional sIgM/D/G but not IgA (Tables 1,2). Using soluble anti-sIgM, small but significant apoptotic effects were observed (Fig. 5), not recapitulated by dysfunctional sIgA. In stark contrast, anti-sIgD signals did not trigger apoptosis and HCs retained cellular integrity (Fig. 5 A). In the absence of additional material from IgD^+ve^ mult-HCL, we examined sIgD mediated cellular responses in a separate IgD^+ve^ single isotype (s)-HCL case. Again, anti-sIgD stimuli did not trigger apoptosis (Fig. 5 B,C), using soluble (Fig. 5 C) or immobilized anti-sIg stimuli (data not shown), revealing isotype specific roles. Consequently, in 2/2 primary HCL samples signaling via sIgD appears to sustain tumor cell integrity, to differ to effects of signal transduction via non-sIgD isotypes in the BCR, and confirm observations in replicate cases. A larger cohort study of IgD^+ve^ mult-HCL cases will be required nevertheless to examine how consistently IgD transduced signals rescue HCs from apoptosis mediated by non-IgD isotypes but it is clear from our data that this potential exists. Importantly, the lack of any observed effects with anti-sIgD also validated assays with other isotypes that did show significant apoptosis, under identical conditions.

## Discussion

This study addressed the intriguing question of a functional role for individual variant sIg isotypes in mult-HCL, in a background of mutant *BRAF* V(600)E, that constitutively activates a critical component of the signaling cascade normally activated by a functional BCR. Such a study is a pre-requisite to address whether the BCR sustains tumor survival *in-vivo* in mult-HCL, and what the nature of the antigen might be that triggers BCR signals.

Variant isotype function on HCs was analyzed by a rigorous strategy that delineated CD19^+^ tumor cells by CD103 and/or CD11c^Hi^ co-expression. Two functionally distinct mult-HCL sets emerged by immunophenotype differing by IgD co-expression (IgD^+ve^ and IgD^−ve^), in which marked differences were apparent in isotype function. In IgD^+ve^ cases, more than one sIgH isotype transduced Ca^2+^ flux with a persistent more robust pattern, whereas in IgD^−ve^ cases only a single switched isotype signaled Ca^2+^ flux of a fast transient nature, despite the presence of other sIgH isotypes. Functional Ca^2+^ flux via specific functional sIgH isotype or sIgL associated tightly with the ability to endocytose BCR, substantiating the linkage of endocytic internalization of the BCR as dependent on functional signaling [3]. Endocytosis of BCR allows B-cells to process and present cognate antigen, and leads to degradation of internalized BCR [26]. Functional observations were reproducibly observed in replicate cases, but will require a wider dissection to establish definitive coherent features with each subset. However, it needs to be recognized that there are restrictions to the size of mult-HCL cohorts that can be examined, given the rare nature of disease.

Functional sIg-mediated Ca2^+^ flux also associated closely with amplification of ERK1/2 phosphorylation in mult-HCL. These cases uniformly harbored a monoallelic *BRAF* V600E mutation, which associates with constitutive activation of ERK1/2 reported in HCL [19], [20]. In normal B-cells, the wild type BRAF activation loop (T^599^VKS^602^) induces kinase activity by conformational changes [27], whereas the V600E mutation intrinsically achieves a constitutively active conformation by mimicking the loop, leading to chronic MEK/ERK signaling and transformation [28]. The mutation bypasses several steps of BRAF activation and renders the oncoprotein resistant to negative regulation [29]. It suggests that in mult-HCL with monoallelic mutant *BRAF* V(600)E, that either the wild type BRAF allele is being activated or that possibly alternative pathways are being recruited by BCR transduced signals to increase constitutive levels of ERK1/2 phosphorylation, as observed in this study. HCL therefore presents a model disease in which to dissect the molecular interplay between mutant B-RAF and wild type A-RAF and C-RAF in transducing BCR-mediated signals to the MAPK pathway. It also suggests that other receptor-ligand interactions have potential to augment phospho-ERK levels in HCs, as signalling conduits for specific tumor requirements.

IgD co-expression correlated with a functional assembly of each variant isotype BCR on single HCs, suggesting that there are no intrinsic steric constraints to multiple sIgH isotypes packaging in the endoplasmic reticulum prior to surface expression. In contrast, in IgD^−ve^ mult-HCL dysfunctional sIgH isotypes were apparent, notably with sIgM which failed to transduce measurable intracellular signals and remained immobilized on the cell surface. It is possible that sIgM or other isotypes in the IgD^−ve^ subset may not be expressed in a fully functional form. However, unless sIgM is fully assembled, it fails to reach the cell surface [30]. The situation with sIgG is less clear, and whether partially assembled and dysfunctional sIgG molecules can be trafficked to the outer cell membrane. Since sIgG is functional in some IgD^−ve^ cases, it would tend to argue that in the IgD^−ve^ subset there appears to be a successive loss of function of co-expressed isotype. Whether IgD^−ve^ cases result from loss of expression of IgD, with subsequent successive loss of function of individual isotypes, remains to be determined. We have previously suggested that individual variant isotypes in mult-HCL arise by a mechanism of differential RNA processing [15], and altered splicing events may underlie such IgD loss. Two mult-HCL cases also displayed dual IgL expression, and in these signals transduced by individual light chain to ERK1/2 phosphorylation were similarly restricted to a single sIgL type.

Notably, our data reveal that BCR mediated signals that lead to apoptosis in HCs carrying mutant *BRAF* are isotype specific, using both soluble and immobilised anti-sIgH stimuli, although due to sample availability of this rare leukemia the latter observations with immobilised ‘antigen’ were restricted, but nevertheless experimentally replicated. These data suggest that in IgD^−ve^ mult-HCL, BCR transduced signals appear not to be relevant to tumor survival. The data also suggest that signals downstream of ERK1/2 phosphorylation bifurcate when initiated via the non-IgD BCR complex (apoptosis) or via *BRAF* V(600)E (pro-survival). They raise the intriguing possibility that acquisition of the *BRAF* V(600)E mutation may be a mechanism to by-pass any BCR signalling constraints in mult-HCL. Constitutively active *BRAF* V(600)E in HCL would also abrogate a requirement for ligand independent tonic signals via the BCR, as these signals are transduced via ERK1/2 phosphorylation [31]. Tonic BCR signals are a requirement in DLBCL [32], but not in FL [33]. *BRAF* V(600)E in HCL clearly mediates pro-survival tumor signals, as targeting the mutant protein with Vemurafenib (VEM) leads to clinical resolution of disease [34]–[36].

BCR signaling in response to antigen in the absence of co-stimuli (CD40:CD40L, TLR ligands, cytokines) is known to lead to activation-induced cell death (AICD) [7]–[9]. This may involve oligomeric assembly of higher-order BCR complex structure that promotes activation but disrupt survival signals [37]. A key question that remains is to what extent B-cell tumors retain the features of the cell of origin or normal B-cell counterpart that has transformed. A role for T-cell dependent antigen activation in origins of HCs in typical disease is as yet not defined. The absence of T-cell derived co-stimuli via this route however is suggested in HCL origins by lack of CD27 expression [1], [25], implicating origins from unusual memory B-cells [38]. SHM imprints are evident in HCs, strongly suggesting a role for antigen in initiating mutations with AID expressed, possibly at extra-follicular sites as T-cell independent events [1], [38]. BCR signals in IgD^−ve^ mult-HCL that lead to apoptosis may then be recapitulating AICD observed in B-cells activated via the BCR in the absence of T-cell help, and tend to support the contention that mult-HCL origins implicate a T-cell independent pathway. Whether co-stimuli such as TLR ligands can rescue the sIgM/G/A signalling cascade to apoptosis in mult-HCL remains to be established.

In HCL, an opposing role of IgD as compared with other isotypes is clearly apparent as sIgD transduced stimuli abrogate pro-apoptotic signals from the BCR, revealing differences in signals initiated by sIgD as compared to other isotypes. They appear to parallel known divergence in IgD and IgM signals in normal B-cells, with the same antigenic specificity. IgD elicits a faster, stronger and more durable signal than IgM whereas only the latter is under control of a negative feedback loop [39]. Despite induction of active Ca^2+^ influx and activated tyrosine kinase signals in both, only anti-IgM antibodies induce cell death, indicative of differing downstream effector pathways [40]. In malignant B-cells however, this difference in IgM and IgD roles is not so readily apparent. In CLL, anti-IgD signals produce different subset responses, in some cases preventing apoptosis above spontaneous levels and in others accentuating them [41]. Apoptotic effects of soluble anti-sIgM in CLL can be abrogated with immobilized anti-sIgM, suggesting that the level of cross-linking impacts on downstream effects in these leukemic cells [42], but not in HCs. In mult-HCL therefore, it is conceivable that sIgD signals may rescue or over-ride pro-apoptotic stimuli by other isotypes on the same HCs, leaving open a role for antigen in tumor persistence in this subset of disease. However, the functional role of sIgD in normal mature B-cells remains largely enigmatic [43], creating some uncertainty whether sIgD signals in mult-HCL may in fact be relevant *in-vivo*. Whether mult-HCL has entirely by-passed any requirements for BCR, possibly due to mutant *BRAF* constitutively activating the ERK1/2 pathway, remains at present speculative.

Mult-HCL presents with unique features of multiple variant sIg isotypes, and in this first study of the individual isotypes in the BCR in this disease these isotypes emerge as differing functionally. Of these, sIgD appears to have a potential role in tumor survival and progression *in-vivo*, although as with normal B-cells, it is as yet not clear whether specific sIgD signals are recruited for mult-HCL survival. In non-IgD expressing mult-HCL, the possibility exists that BCR signals that lead to apoptosis can be exploited to synergise with VEM activity to maximize potency in refractory disease. Targeting specific components of BCR signaling to ablate tumors is already at the clinical application stage in several B-cell malignancies [14], and understanding these pathways in mult-HCL informs both disease immunobiology and may have a therapeutic relevance if VEM refractory disease appears.

## Supporting Information

Figure S1 **Multiparameter flow cytometry to define immunophenotype of single tumor cells in mult-HCL.** Normal PBMNCs and isolated spleen cells from HCL cases were immunophenotyped by gating on live cells and staining for CD19/CD11c/CD103 and sIgH/L surface expression. (A) Following FSC/SSC gating, CD19^hi^CD11c^hi^ cells (for 7/10 cases) or CD19^hi^CD11c^hi^CD103^+^ cells (for 3/10 cases) were examined for sIg expression using rabbit F(ab’)_2_ anti-human Ig antibodies. Normal PBMNC are virtually devoid of B-cells that are dual CD11c^hi^CD103^+^. (B) Representative example of immunophenotype of a mult-HCL tumor. IgD^−ve^ mult-HCL Case 10 CD19^hi^CD11c^hi^CD103^+^ cells display co-expression of sIgG and λ in 100% of HCL cells, with subsets of cells also sIgA (72%) and sIgM (18%) positive.
(TIF)

Figure S2 **Anti-sIg stimulation results in endocytosis of functional BCR in IgD^+^ mult-HCL.** Cells were stimulated with goat F(ab’)_2_ anti-Ig antibodies for 1 hour either at 37°C to allow endocytosis or at 4°C to prevent it. Changes in stimulated sIg expression levels were then measured by secondary staining using rabbit F(ab’)_2_ anti-goat F(ab)_2_ (A, top panels). Changes in expression of paired sIg were measured using rabbit F(ab’)_2_ anti-Ig (A, lower 2 rows). Loss of functional sIg was further confirmed by measuring induction of calcium flux following endocytosis (B). Using the anti-goat secondary antibody method (A, top panels) stimulation via sIgD, λ and sIgM in Case 13 as a representative example resulted in reduction of surface expression of these specific isotypes. Stimulation of non-functional sIgA in this tumor did not result in endocytosis (A, top right panel). Using the rabbit F(ab’)_2_ staining method (A, lower 2 rows) stimulation of sIgD (left panels) in Case 13 resulted in loss of l, but not k light chain expression. Lambda stimulation, second column, resulted in loss of both surface IgD and IgM expression. IgM stimulation, third column, resulted in marginal loss of l but not κ surface expression. Inset of a Table of the sIg phenotype and functional sIg isotypes is included for reference purposes to evaluate data (A, lower right). The ability to induce Ca^2+^ flux (B) via IgD was markedly reduced following endocytosis by λ stimulation (B, left panel). Ability to induce Ca^2+^ flux via λ was ablated by IgD endocytosis and also greatly reduced by IgM endocytosis (B, centre panel). Similarly, IgM induced flux was completely ablated following endocytosis following anti-λ stimulation (B, right panel).
(TIF)

Figure S3 **Stimulation of BCR in mult-HCL triggers apoptosis not proliferation.** Cells were stimulated with goat F(ab`)_2_ anti-Ig antibodies for 4 hours at 37°C and early apoptosis was measured in CD19+CD11c+CD103+ HCL cells using Apostat (left panels). Alternatively, XTT, a tetrazolium salt that is cleaved to formazan in metabolically active cells only was used to assess viability and proliferation following anti-BCR stimuli and OD assayed at 490 nm (right panels). In 2/2 HCL cases (HCL #3, top panels; HCL #10, lower panels) ApoStat assays revealed significant increases in apoptosis in response to both functional heavy (IgG) and light (κ; HCL 3)/λ;HCL 10) chain stimulation. No apparent increase in numbers of metabolically active tumor cells was observed in response to BCR stimulation in either HCL case by XTT assay (right panels).
(TIF)

File S1 **Contains supplementary materials and methods and Table S1.**
(DOCX)
