# Supplementary figures and images for: Variant B Cell Receptor Isotype Functions Differ in Hairy Cell Leukemia with Mutated BRAF and IGHV Genes
Source: PLoS One. 2014 Jan 30;9(1):e86556. doi: 10.1371/journal.pone.0086556 (PMC3907534; doi:10.1371/journal.pone.0086556)

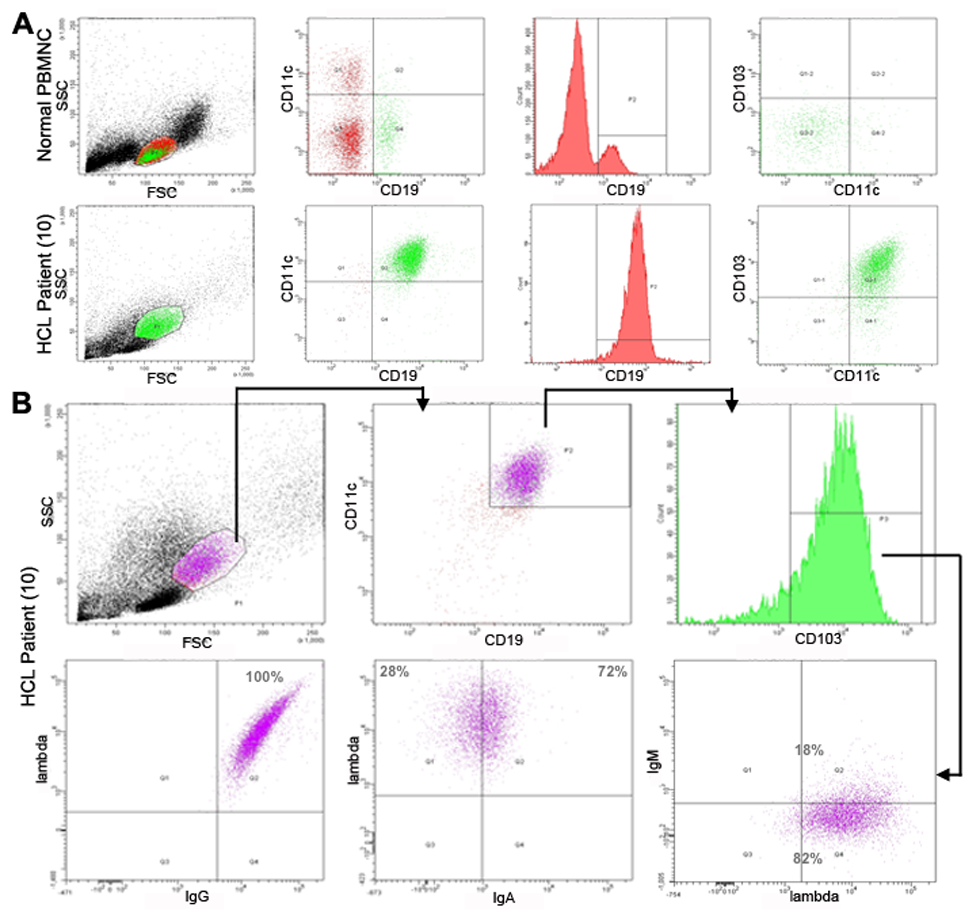

Supplement: Figure S1 — Multiparameter flow cytometry to define immunophenotype of single tumor cells in mult-HCL. Normal PBMNCs and isolated spleen cells from HCL cases were immunophenotyped by gating on live cells and staining for CD19/CD11c/CD103 and sIgH/L surface expression. (A) Following FSC/SSC gating, CD19hiCD11chi cells (for 7/10 cases) or CD19hiCD11chiCD103+ cells (for 3/10 cases) were examined for sIg expression using rabbit F(ab’)2 anti-human Ig antibodies. Normal PBMNC are virtually devoid of B-cells that are dual CD11chiCD103+. (B) Representative example of immunophenotype of a mult-HCL tumor. IgD−ve mult-HCL Case 10 CD19hiCD11chiCD103+ cells display co-expression of sIgG and λ in 100% of HCL cells, with subsets of cells also sIgA (72%) and sIgM (18%) positive. (TIF) [file pone.0086556.s001.tif]

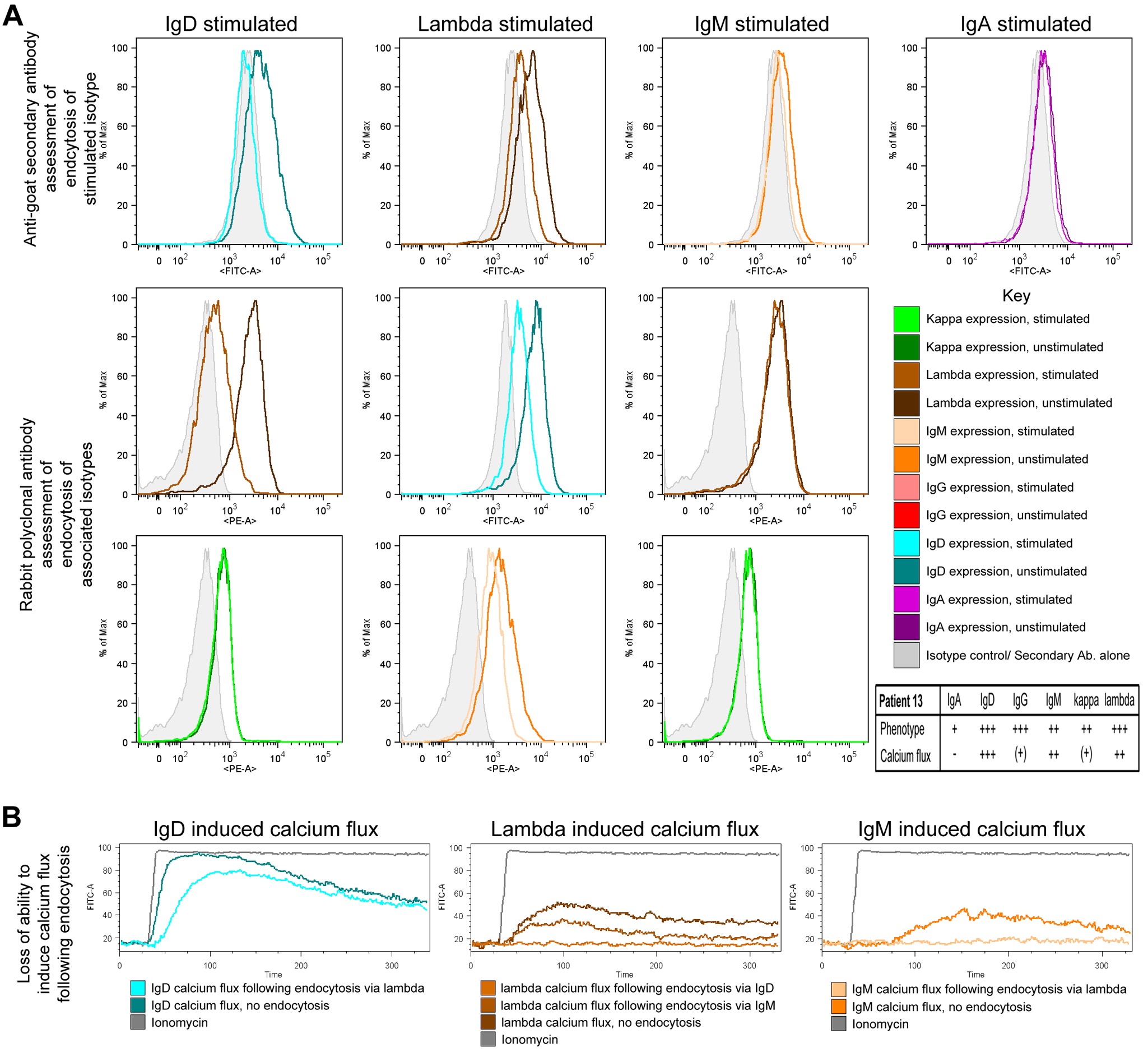

Supplement: Figure S2 — Anti-sIg stimulation results in endocytosis of functional BCR in IgD+ mult-HCL. Cells were stimulated with goat F(ab’)2 anti-Ig antibodies for 1 hour either at 37°C to allow endocytosis or at 4°C to prevent it. Changes in stimulated sIg expression levels were then measured by secondary staining using rabbit F(ab’)2 anti-goat F(ab)2 (A, top panels). Changes in expression of paired sIg were measured using rabbit F(ab’)2 anti-Ig (A, lower 2 rows). Loss of functional sIg was further confirmed by measuring induction of calcium flux following endocytosis (B). Using the anti-goat secondary antibody method (A, top panels) stimulation via sIgD, λ and sIgM in Case 13 as a representative example resulted in reduction of surface expression of these specific isotypes. Stimulation of non-functional sIgA in this tumor did not result in endocytosis (A, top right panel). Using the rabbit F(ab’)2 staining method (A, lower 2 rows) stimulation of sIgD (left panels) in Case 13 resulted in loss of l, but not k light chain expression. Lambda stimulation, second column, resulted in loss of both surface IgD and IgM expression. IgM stimulation, third column, resulted in marginal loss of l but not κ surface expression. Inset of a Table of the sIg phenotype and functional sIg isotypes is included for reference purposes to evaluate data (A, lower right). The ability to induce Ca2+ flux (B) via IgD was markedly reduced following endocytosis by λ stimulation (B, left panel). Ability to induce Ca2+ flux via λ was ablated by IgD endocytosis and also greatly reduced by IgM endocytosis (B, centre panel). Similarly, IgM induced flux was completely ablated following endocytosis following anti-λ stimulation (B, right panel). (TIF) [file pone.0086556.s002.tif]

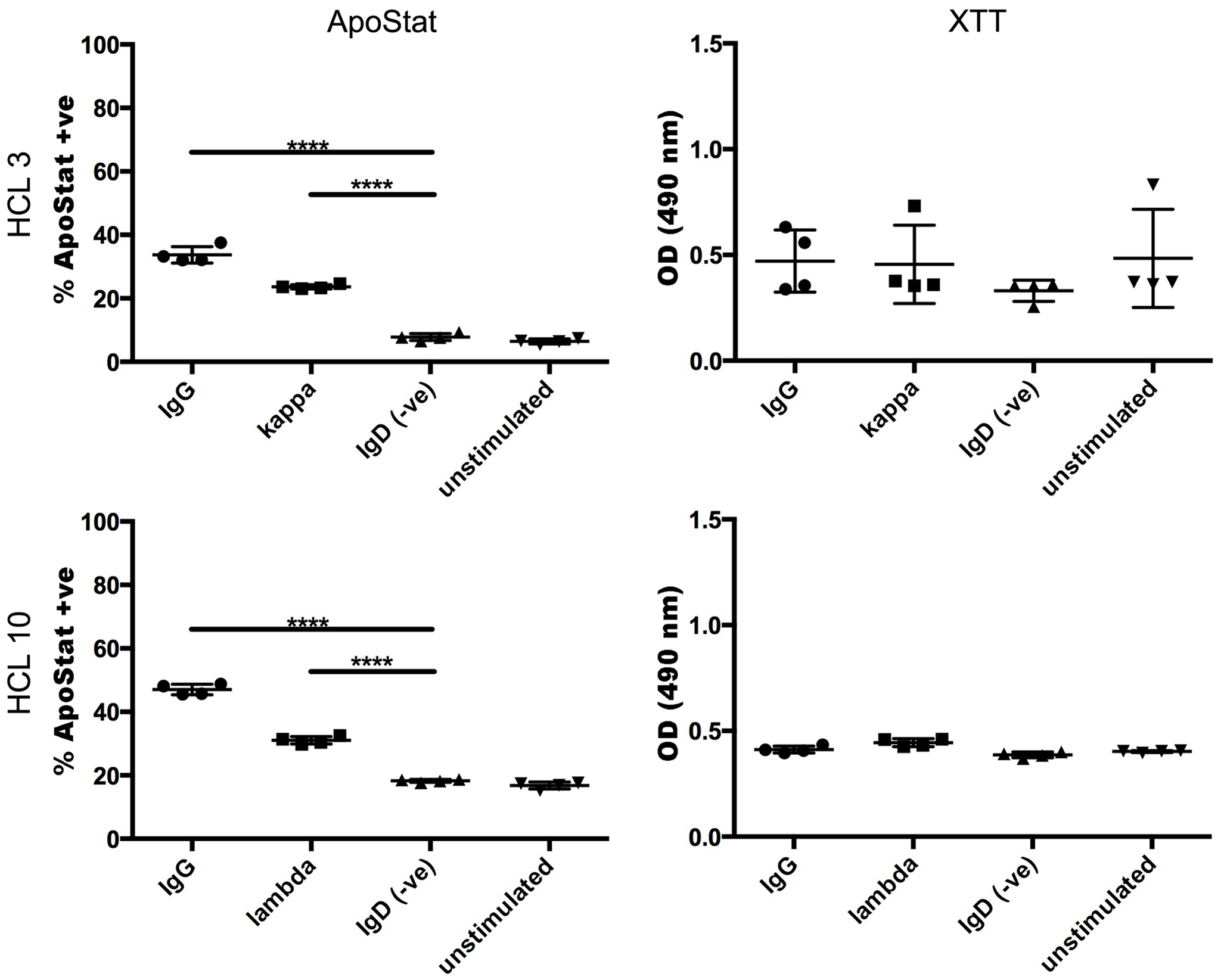

Supplement: Figure S3 — Stimulation of BCR in mult-HCL triggers apoptosis not proliferation. Cells were stimulated with goat F(ab`)2 anti-Ig antibodies for 4 hours at 37°C and early apoptosis was measured in CD19+CD11c+CD103+ HCL cells using Apostat (left panels). Alternatively, XTT, a tetrazolium salt that is cleaved to formazan in metabolically active cells only was used to assess viability and proliferation following anti-BCR stimuli and OD assayed at 490 nm (right panels). In 2/2 HCL cases (HCL #3, top panels; HCL #10, lower panels) ApoStat assays revealed significant increases in apoptosis in response to both functional heavy (IgG) and light (κ; HCL 3)/λ;HCL 10) chain stimulation. No apparent increase in numbers of metabolically active tumor cells was observed in response to BCR stimulation in either HCL case by XTT assay (right panels). (TIF) [file pone.0086556.s003.tif]
