# Supplementary material for: Variant B Cell Receptor Isotype Functions Differ in Hairy Cell Leukemia with Mutated BRAF and IGHV Genes
Source: PLoS One. 2014 Jan 30;9(1):e86556. doi: 10.1371/journal.pone.0086556 (PMC3907534; doi:10.1371/journal.pone.0086556)
Supplement: File S1 — Contains supplementary materials and methods and Table S1. (DOCX) [file pone.0086556.s004.docx]

**SUPPORTING INFORMATION**

**Variant B cell receptor isotype functions differ in Hairy cell leukemia with mutated *BRAF* and *IGHV* genes**

Nicola J Weston-Bell, Francesco Forconi, Hanneke C Kluin-Nelemans & Surinder S Sahota

**Supplementary Materials and Methods**

*Patient Samples*

The diagnosis of typical HCL disease was established on the basis of clinical criteria, morphology of the neoplastic cells in blood and histology of bone marrow and spleen, cytochemical analysis, and immunophenotype (CD11C/CD19/CD22/CD25^+^) [22]. Patients underwent splenectomy, and disaggregated splenic lymphocytes were stored in liquid N_2_ until use.

Frozen samples were thawed, washed and resuspended in complete RPMI medium (cRPMI) (RPMI 1640, 20% fetal calf serum, 1 mM sodium pyruvate MEM, 0.1 mM MEM non-essential amino acids, 2 mM L-glutamine (Gibco Invitrogen, Paisley, UK)) and allowed to stabilise at 37ºC in 5% CO_2_ for at least 1h prior to use. Viablilty was assessed by trypan blue exclusion.

*Immunophenotyping*

For flow cytometry (FC), HCL cells were incubated with α-CD19-perCP-cy5.5, α-CD19-Pacific Blue or α-CD19-V450 (BD Pharmingen, Oxford, UK) and α-CD11c-APC at 1:10 (Biolegend, San Diego, CA, USA) and/or α-CD103-PE-Cy7 at 1:20 (BioLegend, London, UK) with combinations of rabbit F(ab’)_2_ α-IgA-FITC, α-IgD-FITC. α-IgD-PE, α-IgG-FITC, α-IgM-PE, α-kappa-FITC, α-kappa-PE, α-lambda-FITC and α-lambda-PE (all Dako, Ely, UK), or with relevant isotype control antibodies in FACS Buffer (1% BSA, 4 mM EDTA, 0.15 mM sodium azide in phosphate buffered saline) for 30 minutes at 4°C. Isotype control labeling was performed with mIgG_1_,k-perCP-cy5.5 (BD), mIgG_1_,k-APC (BD), mIgG_1_,k-Pacific Blue (BD), mIgG_1_,k-V450, mIgG_1_,k-PE/Cy7 (BioLegend), rF(ab)_2_-FITC (Dako) and rF(ab)_2_-PE (Dako) at equivalent concentration to relevant staining antibody. Cells were gated first as lymphocytes for measurement of tumor population or large lymphocytes for hairy cell gating according to forward and side scatter, then CD19^+^ CD11c^+^ hairy cells were analyzed on a FACSCalibur or FACS Canto II (BD Biosciences, Oxford, UK) for sIg expression. Isotype controls were used to establish correct gates. Data was analyzed using CellQuest Pro or FACSDiva software (BD Biosciences).

*BCR induced intracellular calcium flux*

Approximately 5x10^6^ cells were washed and loaded with 7 µM Fluo-3AM (FluoroPure grade, Molecular Probes, Invitrogen, Paisley, UK) in cRPMI medium with 0.04 % pluronic F-127 (Sigma, Poole, UK) for 30’/37°C. Cells were washed and stained with α-CD19-perCP-cy5.5 at 1:10 or α-CD19-Pacific Blue/V450 at 1:20 (BD Biosciences, Oxford, UK) with α-CD11c-APC at 1:10 for 10 minutes at room temperature in the dark, before a final wash in cRPMI. CD19+CD11c+ gated lymphocytes were acquired in cRPMI using a FACSCalibur or FACS Canto II (BD Biosciences, Oxford, UK) for 30 seconds unstimulated, before unlabeled goat F(ab’)_2_ α-human IgA, IgD, IgG, IgM, kappa or lambda (Southern Biotech) was added at 20 µg/ml and cells acquired for the remainder of 5 minutes. Each of these stimulating antibody preparations is formulated with the same buffers, and used at identical concentrations in control assays with non-functional isotypes. Alternatively, to confirm that CD19/CD11c/CD103 gating antibodies did not trigger calcium flux themselves, α-CD19-perCP-cy5.5 at 1:10, α-CD19-Pacific Blue/V450 at 1:20, α-CD11c-APC at 1:10 or α-CD103-PE-Cy7 at 1:20 were added, following 30s unstimulated, and cells acquired for the remainder of 5 minutes. Data was analyzed using FlowJo (Tree Star Inc., Ashland, OR, USA).

*BD Phosflow protocol for measurement of BCR induced ERK phosphorylation*

BD Phosflow protocol II (mild alcohol permeabilization) with BD Cytofix and ice cold 90% MeOH was used. 1x10^6^ cells in 100µl cRPMI were stimulated with 20 µg/ml goat F(ab’)_2_ anti-human IgA, IgD, IgG, IgM, kappa or lambda and stopped by fixation after 30, 20, 15, 10, 7, 4 and 2 minutes. Cells were fixed for 15 minutes at 37ºC then immediately transferred to -80ºC for storage overnight. Following thawing cells were pelleted and resuspended in 2 ml ice cold 90% methanol and incubated for 20 minutes at 4ºC to permeabilize. Cells were then washed twice in FACS buffer and stained with α-CD19-perCP-cy5.5 at 1:10 or α-CD19-Pacific Blue/V450 at 1:20 (BD Biosciences, Oxford, UK) with α-CD11c-APC and α-phosphoERK1/2-alexafluor488 at 1:20 (BD). Data were acquired using a FACSCalibur or FACS Canto II (BD Biosciences) and CD19+CD11c+ gated lymphocytes analyzed for changes in phosphoERK fluorescence in CellQuest Pro or FACSDiva software.

*BCR endocytosis*

Two methods were employed to assess changes in surface Ig expression following stimulation. For both methods goat F(ab)_2_ α-human IgA, IgD, IgG, IgM, kappa or lambda antibodies (Southern Biotech) were allowed to bind for 30 minutes at 4ºC in complete RPMI. Cells were then washed and incubated for 1 hour at 37ºC in complete RPMI to allow endocytosis or at 4ºC in endocytosis stop buffer (0.1% azide, 0.1% BSA in PBS). Cells were then washed in cold endocytosis stop buffer and stained with α-CD19-V450 (BD) and α-CD11c-APC (BD). At this point, two methods of measuring changes in sIg expression were employed in parallel. Either cells were stained with a FITC-labeled rabbit F(ab’)_2_ anti-goat F(ab’)_2_ (Jackson Immunology, where, where) secondary antibody to detect remaining goat F(ab’)_2_ on the cell surface, or with rabbit F(ab’)_2_ α- human Ig antibodies, as for immunophenotyping (Dako) for 30 minutes at 4ºC to allow analysis of changes in paired IgH and IgL chains. Data were acquired on a FACS Canto II with FACSDiva (BDBiosciences) and FlowJo (Treestar) software for analysis.

*Induction of intracellular calcium flux following BCR endocytosis*

Endocytosis of sIg was induced as above, with 30 minutes pre-binding of goat F(ab)_2_ anti-IgH or IgL (Southern Biotech), followed by 1 hour incubation at 37ºC in cRPMI. Cells were then washed and loaded with fluo3-AM for 30 minutes at 37ºC, followed by staining with α-CD19-V450 and α-CD11c-APC. Data were acquired as for calcium flux method above using a FACS Canto II and analysed in FlowJo (Treestar).

*Induction of Apoptosis following BCR stimulation*

Stimulations were performed in 96-well plates in quadruplicate. 0.1-0.2x10^6^ HCL cells were stimulated with 20 µg/ ml goat F(ab)_2_ α-human IgA, IgD, IgG, IgM, kappa or lambda soluble antibodies or 10-20 µg/ml anti-human IgA, IgG or IgM coated immunobeads (Irvine Scientific, Newtownmountkennedy, Ireland) in cRPMI for 2/3 hours/37ºC [100 ul medium + 4 μl Ab, diluted 1/25]. Induction of apoptosis was measured either using Apostat (R&D Systems, Abingdon, UK), a cell permeable FITC-conjugated pan-caspase inhibitor that irreversibly labels cells undergoing apoptosis, or Apo2.7-PE (Beckman-Coulter, High Wycombe, UK), a mitochondrial membrane protein which is exposed on cells undergoing early apoptosis. Apostat was added according to manufacturer’s protocol, 30’ prior to the end of the incubation time, Apo2.7-PE (1:10) staining was performed together with CD19 and CD11c staining. At the end of incubation times cells were washed with FACS buffer and stained with α-CD19-V450 (BD) or α-CD19-APC-H7 (BD) with α-CD11c-APC (BD)/α-CD103-PE-Cy7 (BioLegend), with Apo2.7-PE as appropriate, for 10 minutes at room temperature and washed twice with FACS buffer. Cells were then analyzed using a FACS Canto II with FACSDiva and PRISM (GraphPad, La Jolla, CA, USA) software.

*Analysis of proliferation following BCR stimulation*

0.1-0.2x10^6^ HCL cells were stimulated in 96-well plates in quadruplicate with 20 µg/ ml goat F(ab)_2_ α-human IgA, IgD, IgG, IgM, kappa or lambda in cRPMI at 37ºC and Cell Proliferation Kit II (XTT) (Roche, Welwyn Garden City, UK) was used to measure proliferation according to the manufacturer’s protocol. Briefly, XTT, a tetrazolium salt that is cleaved to formazan in metabolically active cells only, and PMS, an electron coupling reagent, were included for 4 hours alongside anti-BCR stimulation, then OD measured at 490 nm. Data were analyzed in PRISM (GraphPad, La Jolla, CA, USA).

|  |  |  | | | | | |  | | | | | | **IGHV gene status** | | | | | | |
| --- | --- | --- | --- | --- | --- | --- | --- | --- | --- | --- | --- | --- | --- | --- | --- | --- | --- | --- | --- | --- |
|  |  | **HCL phenotype** | | | | | | **sIg phenotype** | | | | | |  |  | **clonal isotypes** | | | |  |
| **sIg Status** | **Case** | CD19 | CD11c | CD27 | CD103 | % tumor | BRAF V600E | **A** | **D** | **G** | **M** | **K** | **L** | **IGHV** | **% homol** | **A** | **G** | **M** | **D** | **ICH** |
| **D-ve** | **2** | **+** | **+** | ND | **+** | 86 | + | **-** | **-** | **+++** | **++** | **+++** | **-** | 3-30 | 96.7 | + | + | ND | - | **-** |
| **D-ve** | **3** | **+** | **+** | **-** | **+** | 83 | + | **-** | **-** | **+++** | **++** | **+++** | **-** | 3-30 | 96.1 | + | + | + | - | **-** |
| **D-ve** | **4** | **+** | **+** | ND | ND | 73 | + | **-** | **-** | **+++** | **++** | **+++** | **-** | 1-02 | 93.1 | - | + | + | - | **+** |
| **D-ve** | **5** | **+** | **+** | **-** | **+** | 80 | + | **+++** | **-** | **+++** | **+** | **++** | **+++** | 3-15 | 92.2 | + | + | ND | - | **+** |
| **D-ve** | **10** | **+** | **+** | **-** | **+** | 55 | + | **++** | **-** | **+++** | **+** | **-** | **+++** | 1-46 | 95.1 | + | + | + | + | **+** |
| **D-ve** | **11** | **+** | **+** | **-** | **+** | 52 | + | **+** | **-** | **+++** | **+** | **+++** | **-** | 3-20 | 94.8 | - | + | + | - | **-** |
| **D+ve** | **6** | **+** | **+** | **-** | **+** | 82 | + | **+** | **+++** | **++** | **+** | **++** | **+++** | 5-51 | 98.6^#^ | + | + | + | + | **+** |
| **D+ve** | **7** | **+** | **+** | **-** | **+** | 86 | + | **-** | **+++** | **+++** | **+++** | **+++** | **-** | 3-23 | 96.5 | + | + | + | + | **+** |
| **D+ve** | **9** | **+** | **+** | **-** | **+** | 65 | + | **-** | **+++** | **+++** | **+++** | **+++** | **-** | 3-30 | 92.7 | + | - | + | + | **-** |
| **D+ve** | **13** | **+** | **+** | **-** | **+** | 67 | + | **+** | **+++** | **+++** | **++** | **++** | **+++** | 3-33 | 96.2 | + | + | + | + | + |

**Table S1. Summary of immunophenotype of mult-HCL cases and mutation status of *BRAF* and *IGHV* genes^*^**

**^*^**BRAF mutation was confirmed as T1860A-V600E; Clonal isotype variant transcripts were identified using our reported procedure [15,16]; ^#^<2% mutation deviation from germline can be considered for functional imprint of antigen selection and impact of somatic hypermutation [Forconi F, Sahota SS et al Leukemia 2004;18:882-3]; ICH denotes intraclonal heterogeneity in IGHV gene sequences; 10/10 tumors are mult-HCL, percentage of hairy cells expressing each sIg isotype is represented as: - <10% cells, + 10-50% cells, ++ 50-90% cell, +++ >90% cells; CD19^+^CD11c^+^CD103^+^ or CD19^+^CD11c^+^ (Patient 4) were used to delineate tumor burden.
